# MicroRNAs in the Evaluation and Potential Treatment of Liver Diseases

**DOI:** 10.3390/jcm5050052

**Published:** 2016-05-10

**Authors:** Amar Mahgoub, Clifford J. Steer

**Affiliations:** 1 Department of Medicine, Division of Gastroenterology, Hepatology and Nutrition, University of Minnesota Medical School, Veterans of Foreign Wars Cancer Research Center, 406 Harvard Street, S.E., Minneapolis, MN 55455, USA; 2 Department of Genetics, Cell Biology and Development, University of Minnesota Medical School, Veterans of Foreign Wars Cancer Research Center, 406 Harvard Street, S.E., Minneapolis, MN 55455, USA

**Keywords:** acute liver failure, biogenesis, epigenetics, hepatitis, hepatocellular carcinoma, liver diseases, liver regeneration, metabolic liver diseases, microRNAs, NAFLD, NASH, personalized medicine, non-coding RNAs, partial hepatectomy, systems biology

## Abstract

Acute and chronic liver disease continue to result in significant morbidity and mortality of patients, along with increasing burden on their families, society and the health care system. This in part is due to increased incidence of liver disease associated factors such as metabolic syndrome; improved survival of patients with chronic predisposing conditions such as HIV; as well as advances in the field of transplantation and associated care leading to improved survival. The fact that one disease can result in different manifestations and outcomes highlights the need for improved understanding of not just genetic phenomenon predisposing to a condition, but additionally the role of epigenetic and environmental factors leading to the phenotype of the disease. It is not surprising that providers continue to face daily challenges pertaining to diagnostic accuracy, prognostication of disease severity, progression, and response to therapies. A number of these challenges can be addressed by incorporating a personalized approach of management to the current paradigm of care. Recent advances in the fields of molecular biology and genetics have paved the way to more accurate, individualized and precise approach to caring for liver disease. The study of microRNAs and their role in both healthy and diseased livers is one example of such advances. As these small, non-coding RNAs work on fine-tuning of cellular activities and organ function in a dynamic and precise fashion, they provide us a golden opportunity to advance the field of hepatology. The study of microRNAs in liver disease promises tremendous improvement in hepatology and is likely to lay the foundation towards a personalized approach in liver disease.

## 1. Liver Disease and MicroRNAs

### 1.1. The Liver in Precision Medicine

The liver’s enormous regenerative capacity in the face of acute and chronic injury has been recognized and studied for decades, both in humans and animal models. It is the only organ capable of regeneration and healing in a complex fashion that allows it to return to baseline functional capacity including, regulation of metabolism, synthesis, storage and redistribution of nutrients, detoxification, as well as immune modulation [1]. Whether we are studying the liver’s response to external insults, or the normal physiologic repopulation of hepatocytes, the liver provides a unique opportunity to understand the exact mechanisms involved, and their signaling, interaction and feedback pathways associated with such a complex process [2].

Animal models have provided essential knowledge about the liver’s basic physiology and regenerative capability. The earliest of these models is the commonly used rodent model of surgical partial hepatectomy (PH) by Higgins and Anderson, in which two thirds of the liver (70%) was surgically removed, followed by regeneration of the remaining tissue until the original liver mass was restored in ~1 week after resection [3,4]. In addition, liver organogenesis, and liver cell differentiation from progenitor cells have been well characterized [2,4]. These factors allow the liver to be a remarkable system to elucidate epigenomic pathways, and discovery of potential biomarkers that can shed some light on disease states and host response. miRNAs have received a lot of attention in this regard as they are involved in almost every aspect of liver health and disease as well as possessing unique properties of stability and abundance [5].

Despite the great advances in the field of hepatology including diagnostics and therapeutic options, it is obvious that liver disease leads to significant mortality and morbidity, as well as high financial burden. This is especially true when dealing with chronic liver disease such as alcoholic liver disease, chronic viral hepatitis, non-alcoholic liver disease (NAFLD), cirrhosis and hepatocellular carcinoma. As our understanding of these conditions has improved, it’s clear there are multiple points during these chronic conditions when intervention may modify disease progression and avoid significant mortality and morbidity. Interestingly, and as highlighted earlier, not every individual with the disease will develop the dreaded long-term complications.

Clinically, providers still face a number of challenges related to diagnosis, management and follow-up of patients with liver disease. For example, it is not entirely clear how to identify at-risk individuals within a specific population who may later develop progressive disease. Recent studies estimate an NAFLD prevalence of 20% in North America; however, approximately 26% of that population have evidence of steatohepatitis and risk of progression to more advanced disease including cirrhosis, hepatocellular carcinoma, liver failure, death and/or liver transplantation [6]. Another challenge is identifying individuals with acute liver failure who may recover spontaneously *versus* those who will not and may require transplantation. In addition, we are lacking accurate predictors of response to therapy in individuals receiving therapy for chronic viral hepatitis, such as hepatitis C virus, relative to the viral genotype and degree of injury present in the liver. Finally, in individuals receiving liver transplantation, it remains difficult to predict their long-term outcomes in relationship to disease recurrence and response to immunosuppression.

Most of these challenges can be addressed if adequate tools are available that provide accurate predications of response and outcomes; and in essence a personalized care model and approach founded on rigorous science. MicroRNAs are poised to be a critical tool in this model; and are likely to foster the growth of personalized medicine, with the potential to revolutionize how and when care is delivered. The exponential growth in the field of miRNA research, as documented by the number of published studies over the last 15 years, is a testament to the crucial role they play in a almost all aspects of health and disease states [7].

### 1.2. One Diseased Organ, Many Manifestations; Appreciating the Liver’s Uniqueness

The concept of personalized medicine dates back many centuries; it was Hippocrates who noted, “it’s far more important to know what person the disease has than what disease the person has” [8]. It has long been recognized that individuals with similar symptoms may have very different diseases; and those with the same disease would have different manifestations of signs, symptoms, course, outcomes and response to therapy. These observations form the foundation for the “practice” and “art” of medicine, and our ability to combine available knowledge, diagnostic technologies and personal expertise to accurately diagnose, prognosticate and manage a disease. To ensure the optimal outcome one would have to account for not only the nature of the disease, but also the interaction between the unique patient’s genetic profile and environmental factors. Ideally, tailored therapy is decided, and based on the above factors with accurate prediction of outcomes, rather than a trial and error approach. This approach takes into account the individual’s characteristics, preferences and needs at the different stages of a disease from prevention, diagnosis, treatment and follow-up [9].

Many terms exist describing the idea of personalized medicine including precision medicine, targeted medicine and pharmacogenomics. They generally address the concept of “the right patient with the right drug at the right dose at the right time” [9,10]. As we are living in an increasingly diverse world where the classic boundaries between populations, ethnic groups and cultural backgrounds are slowly dissipating the approach of one-size fits all will no longer be applicable. Some early examples of personalized medicine include the first known blood compatibility test used by Reuben Ottenberg in 1907 to avoid hemolytic transfusion reaction; and the identification of polymorphism in the cytochrome P450 2D6 in 1977, which led some patients being treated for hypertension with debrisoquine to experience intense side effects [10].

More recently, a number of national and international entities have come up with specific definitions of personalized medicine in order to standardize the approach, and facilitate for regulations of the field. For example: The President’s Council of Advisors on Science and Technology defines it as “The tailoring of medical treatment to the individual characteristics of each patient”; the Personalized Medicine Coalition uses the definition of “The use of new methods of molecular analysis to better manage a patient’s disease or predisposition to disease”; and the National Cancer institute, NIH notes that it is “A form of medicine that uses information about a person’s genes, proteins and environment to prevent, diagnose and treat disease” [10,11].

The field of medical oncology has led the way with application of personalized medicine with improved understanding of molecular carcinogenesis, patient’s genetic makeup, and pharmacogenomics [12]. The identification of several molecular biomarkers has led to better diagnosis, treatment, response monitoring and prevention in cancer patients, which has revolutionized an ever-evolving field [13]. These biomarkers include BCR-ABL in chronic myeloid leukemia, BRCA 1 and 2 for breast cancer, and BRAF for melanoma [12,13]. The emergence of the area of systems biology has facilitated transitioning personalized medicine from theory to practice. Systems biology is the computational and mathematical modeling of complex biological systems to better understand and predict the phenotypical outcomes. The field includes metabolomics, genomics, proteomics, transcriptomics, and epigenomics, which collectively are called omics [14].

Epigenetic biology initially introduced by the biologist and geneticist Conrad Waddington, aims to explain that observed phenotypes are the results of causal interactions between genes and their products. This term has more recently evolved to recognize the reversibility of interaction mechanisms and the adaptive nature in response to environmental stimuli. Well-recognized epigenetic modifications include chromatin remodeling, DNA methylation, histone modification and microRNA function. The study of epigenetic phenomenon has gained great momentum recently due to technological advances, which led to improved understanding of complex systems [15,16,17,18]. Most notably, microRNAs (miRNAs) have emerged as key players involved in the interaction between the genome and the environment, both globally and at the cellular level, and have provided a wealth of information about development and progression of complex disorders and diseases. In addition, miRNA profiling allows for their use both as biomarkers and potential therapeutic targets, further making the concept of personalized medicine a reality [19,20].

## 2. MicroRNAs: Biogenesis and Regulation

It was not until recently that sufficient evidence became available supporting that the majority of human genome is both transcribed and metabolically active. Specifically, our understanding of non-coding RNA has changed significantly based on recent advances in molecular biology; and the role of RNAs is recognized to include more than a link between DNA code and synthesized proteins [21,22]. Broadly speaking, non-coding RNAs are divided into (i) transcription RNAs (including both tRNA and rRNA); (ii) small RNAs, which are further subdivided into siRNAs, miRNA, snoRNA, snRNAs; and (iii) most recently, long non-coding RNAs [23,24]. As we focus more on miRNAs, it is worth highlighting the concept of RNA silencing—a phenomenon whereby a cell uses double-stranded RNAs (fully or partially) as intermediates to repress expression of their target genes in a sequence-specific manner [18]. Therefore, miRNAs are viewed as providing fine- tuning of transcription depending on environmental factors [12].

MicroRNAs are single-stranded non-coding RNAs that are typically 18–25 nucleotides (nts) in length, and are best known for their role in the post-transcriptional regulation of gene expression. They are the most abundant class of small endogenous non-protein coding RNAs. miRNA genes, which are well conserved, are one of the large gene families found in viruses, plants and animals. In humans, more than 2500 miRNAs have been identified and catalogued [25,26,27]. The majority of miRNA sequences in humans are typically transcribed by introns of non-coding and coding transcripts, with few transcribed by exonic regions. As multiple miRNA loci are located close to each other, the same cluster of regional proximity may be transcribed in a group fashion, however, different miRNAs can be further modulated at the post-transcription level [28,29].

**Figure 1:**
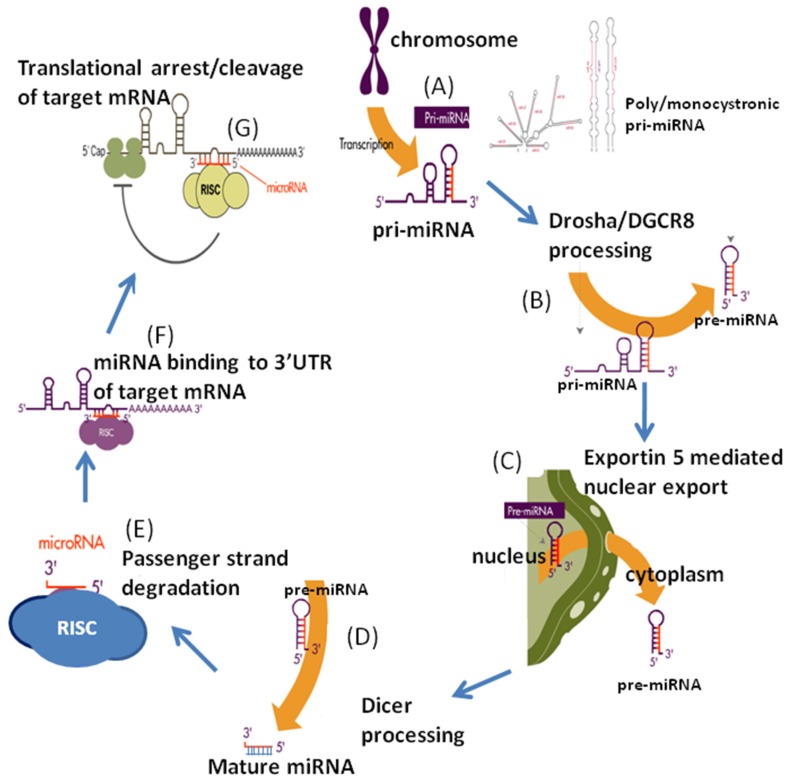
Biogenesis of microRNAs. (**A**): Transcription of primary microRNAs (pri-miRNAs) by RNA polymerase II in the nucleus; (**B**): Pri-miRNA is processed by Drosha and DGCR8 to form a precursor-miRNA (pre-miRNA) approximately 70 nucleotides in length; (**C**): Pre-miRNA is exported to cytoplasm by exportin 5; (**D**): Final processing of pre-miRNA to mature duplex miRNA by RNase enzyme Dicer; (**E**): Incorporation of mature duplex into RNA-induced silencing complex (RISC) where miRNA strand is selectively degraded; (**F**): Binding of complex to the target mRNA guided by mature miRNA; (**G**): Negative regulation of protein translation or degradation of the mRNA transcript based on complementarity of the miRNA to the target sequence. Reprinted with permission from “MicroRNAs as Gatekeepers of Apoptosis”. (*J. Cell. Physiol.* **2010**).

MicroRNA genes are typically transcribed by polymerase II or III, which initially leads to the generation of primary miRNAs (Pri-miRNAs), which usually contain sequences for multiple miRNAs and can be hundreds of nts in length (Figure 1). This structure is then processed and cleaved by Drosha-DGCR8 complex, resulting in the formation of a hairpin-shaped stem-loop structure, which is known as the precursor miRNA (pre-miRNA), and typically around 70 nts in length. The pre-miRNA then is exported outside of the nucleus primarily by exportin 5. Further processing takes place in the cytoplasm by Dicer1-TARBP2, which is an RNase III enzyme, resulting in a two-stranded duplex of miRNA-miRNA*. Typically, it is 18–25 nt long, with one strand designated the guide strand and the other is the passenger strand. Finally the guide strand is incorporated into the RNA-induced silencing complex (RISC), which is a large multiprotein miRNA ribonucleoprotein complex that is the effector compound in modulating target gene transcription. This complex preserves the stability of the mature miRNA in which both ends of the strand are protected by argonaute (AGO) proteins [30,31,32]. Alternative pathways have recently been described that are Drosha-DGCR8 independent as well as Dicer independent, and are likely to greatly advance our understanding of miRNA biogenesis and involvement in states of disease and health [30,32,33]. The miRNA binding sites are located predominantly in the 3′ untranslated region of messenger RNA (mRNA). miRNA exert it’s effect by guiding the RISC complex to the area of complementarity by base pairing on mRNA 3′ region, which in turn will lead to the AGO protein complex to exert its effect by recruiting factors that lead to repression of translation [34].

The two main aspects to consider in the regulation of miRNAs synthesis and processing are (i) epigenetic and (ii) transcriptional control factors. From an epigenetic aspect, miRNA promoters are either located within the introns and exons of the primary gene and therefore, would share similar transcriptional controls or individual promoters. This is particularly true if the promoters are located in inter-genic regions. There are still gaps in knowledge regarding the complex regulatory mechanisms of miRNAs, however the field is advancing rapidly [35,36,37]. Further, it has been shown that a number of nuclear receptors are involved in the transcriptional regulation of miRNA expression, including the small heterodimer partner (SHP) and farnesoid X receptor (FXR). For example: miR-29a promoter activity was increased when FXR agonists were used in animal models [38,39]. In general, miRNAs are detected as (i) extracellular circulating miRNA bound to different lipoproteins; (ii) part of a non-membrane ribonucleoprotein complex, associated with Argonaut proteins; and (iii) contained in exosomes of extracellular vesicle, where exosomes are nano-sized transporters which are involved in communication between neighboring cells [40].

One of the challenges encountered when working with miRNAs is they have been isolated from a variety of tissues, and bodily fluids including saliva, serum as well as stool. For example, the upregulation of fecal microRNAs 223 and 451 are being studied as biomarkers for the presence of colorectal cancer [41]. In addition, different methods are employed to detect miRNAs and their levels. Therefore, there is some heterogeneity in results without a strict standardization of detection tools [42,43]. Currently, most available data have been generated utilizing microarrays with limited sets of probes, which certainly would not account for all miRNAs that are potentially related to disease [43]. Multiple commercially available miRNA detection methods utilizing qPCR are now available, although limitations exist in part due to miRNA-specific primers. More recently, an open source miRNA specific qPCR method has become available, miQPCR, in order to meet some of these challenges [44].

**Figure 2:**
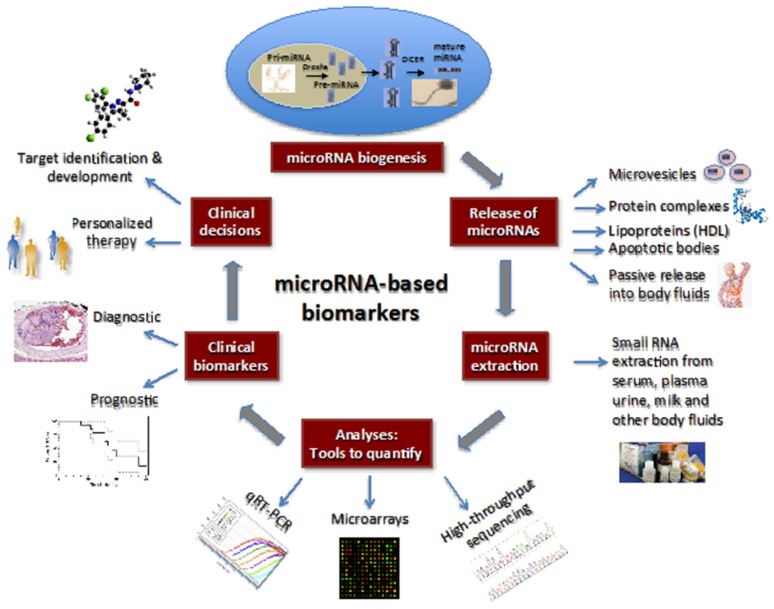
miRNA-based biomarkers. The schematic diagram shows the biogenesis of miRNAs and their secretion into body fluids through various mechanisms, which include mirovesicles, protein complexes, lipoproteins, apoptotic bodies, and passive release. The secreted miRNAs can be extracted with small RNA extraction kits and analyzed for the presence of mature miRNAs with different quantitative approaches. The profile of miRNA populations is used to determine the diagnosis and/or prognosis of the patient’s clinical disorder. On the basis of diagnostic and prognostic utilities, a clinical decision can be made for the targeted and personalized treatment of the disease. Reprinted with permission from “Circulating MicroRNAs as Biomarkers: A New Frontier in Diagnostics” (*Liver Transplant.* **2012**).

It has been well documented that miRNA expression, and detectable levels vary significantly both in liver tissue and serum depending on the conditions of the liver. The relative abundance of miRNAs is influenced directly by disease stage, dietary changes, and genetic manipulations in experimental models [45,46]. Fortunately, circulating endogenous miRNAs are very stable relative to RNAs in general and, therefore detection of aberrant miRNA profiles are the basis for their potential utility as a biomarker reflecting accurate and timely cellular changes (Figure 2).

It is now well established that miRNAs play critical roles in numerous functions involving both cellular and organs growth, proliferation, differentiating, apoptosis, and metabolism. In humans, best estimates suggest that as much as 60%–70% of all genes are either directly or indirectly regulated by miRNAs. It is no easy task to elucidate the precise mechanisms involved for any one particular disease state. However, significant progress has been made, especially in the areas of hepatic malignancy, and other common liver pathologies such as alcoholic liver disease, NAFLD and chronic viral hepatitis. Table 1 provides a summary of sample studies evaluating numerous miRNA changes with target validation. Prior to an elaborate discussion of specific disease states, it is best to review the role of miRNAs in normal liver function as well as observed changes during natural homeostasis.

## 3. MicroRNAs in Normal Liver Development and Maturation

MicroRNAs are some of the key players involved in almost every aspect of liver development and maturation. In hepatic development, the endodermal epithelium of the embryonic foregut will eventually produce hepatoblasts, which are the hepatic progenitor cells. These cells differentiate to give rise to cholangiocytes and hepatocytes. This differentiating process continues from approximately week 6 in the human fetus until birth [18]. Data suggest that some of these progenitor cells will be present through adulthood and form the basis for liver cell replacements during natural liver maintenance activities [47].

Data exist showing specific changes in miRNA levels that correlate with the transition of embryonic stem cells to definitive endoderm, which eventually transition to hepatoblasts in the developing liver. The up- and down-regulation of miRNAs is a time dependent phenomenon and a single miRNA might be up-regulated at a specific stage of development but down-regulated later [48,49]. For example, miR-20b and miR-146a are down-regulated during progenitor cell differentiation into hepatocytes, whereas miR 30a, miR-122, miR-143, and miR-542-5p are increased in their expression levels. In addition, during embryogenesis it is not surprising that a number of miRNAs are increased including, let-7 family, miR-22, miR-99a, miR-122, miR-125b and miR-192, whereas miRNAs 19b, 23a, 92a, 106, 125a and 127 are reduced in levels of expression [49].

Additional studies have shed important light on the interaction of miRNAs and their potential targets as well as their tissue specificity. As an illustration, miR-122 is one of the most abundant and liver specific miRNAs that becomes detectable early in embryogenesis and continues to be highly expressed into adulthood. By using a variety of miR-122 knockdown, knockout, and overexpression mouse models, a number of gene targets for this single miRNA have been identified. As an example, Cutl1 gene is negatively influenced by miR-122 [50,51].

Multiple studies have clearly illustrated the crucial role miRNAs play in development whether by targeting the mature mRNA itself or some of the key players in miRNA maturation in a negative feedback mechanism [52]. Additional studies evaluated differentiation of both human embryonic stem cells and mesenchymal stem cells from umbilical cord into hepatocytes and determined that several miRNAs were either up-regulated (miRNAs 10a, miR-122, and miR-143) or down-regulated (miR 20b, miR-30a and miR-146a) for expression. As the results were not consistent in the studies, it is postulated that the origin of the progenitor cell certainly influences the miRNA profile [48,53].

## 4. MicroRNAs and Liver Proliferation

### 4.1. Hepatic Mass Maintenance

As with any other organ, the liver requires basic mechanisms that allow for replenishment of lost hepatocytes under normal conditions. Furthermore, the liver is the only organ that is capable of regeneration after massive injury. It is becoming more obvious that there is not a single type of cell that is responsible for this process. Rather, a number of potential hepatic progenitor cells have been identified and studied to gain a better insight into the liver’s regenerative process. From animal models, it was traditionally believed that oval stem cells contributed directly to liver regeneration, however recent evidence expands liver regeneration beyond these cells [54]. More recent studies suggest that under certain physiological conditions mature hepatocytes are able to trans-differentiate into cholangiocytes [55]. Other studies have highlighted that peri-central hepatocytes replicate at a faster rate than those in the peri-portal region. It addition, it appears that they are the only hepatocyte population expressing genes activated by the Wnt signaling pathway, suggesting an important role for this population of cells in contributing to maintenance of the hepatocyte population [56]. In addition, it has been shown that in response to different injury models, the liver can induce different hepatic micro-compartments to activate potential progenitor cells. These include, but are not limited to junction of the hepatocyte bile canaliculi and the proximal biliary ductules at the canals of Hering; within the intralobular bile ducts; peripheral to the bile ducts; and within the hepatic parenchyma. Each of these subpopulations of hepatic cells contributes towards maintenance of normal hepatic mass depending on the nature of injury and rate of cell death [57].

The general underlying principals of liver repair and regeneration are (i) it is a well orchestrated process with clear starting and terminating points; (ii) it involves a number of metabolic and epigenetics changes; and (iii) wound healing process may be defective and can therefore lead to diseased states such as increased fibrosis. In general, liver injury followed by repair is achieved in the form of regeneration. Injury may take on many forms including surgical resection, acute and stressful events such alcoholic hepatitis, acute viral hepatitis or ischemic injury; as well chronic injury such as chronic viral hepatitis, chronic heavy alcohol consumption and NAFLD. Regeneration is the physiologic response to these stressors and it is a tightly controlled process involving well-synchronized effectors such as cytokines, growths factors, metabolic changes, progenitor cells; as well as precise interaction between the different liver cells including, hepatocytes, Kupffer cells, endothelial cell, macrophages and stellate cells. There are observed metabolic, genetic and epigenetic changes, which are tightly controlled [58,59,60].

Depending on the degree and duration of injury, regeneration may take on the form of either compensatory hypertrophy (increase in size of cells) or regeneration/proliferation where hepatocytes divide and increase in number and volume, and eventually reaching the appropriate volume to weight ratio which somehow signals termination of regeneration [60]. It is important to note the distinction between compensatory hypertrophy and true regeneration. After PH, the liver does not undergo true regeneration, which typically means regrowth of lost lobes, but rather it undergoes compensatory hypertrophy, *i.e.*, growth of the remaining hepatic tissue.

The liver’s response to injury is healing and regeneration. Regeneration is a tightly controlled process that involves a number of signals and factors between genetic code and the environment. It has the distinct phases of priming where hepatocytes prepare for division by moving from the G0 phase to G1 phase of the cell cycle; proliferation in which hepatocytes either increase in size alone or increase in size followed by division; and termination where a balance between apoptosis and proliferation is established and regeneration is halted once the specific liver volume to body weight ratio is reached [61,62,63].

In animal models, PH has provided the most data regarding the changes observed of key regulatory players including tumor necrosis factors, IL-6, hepatocyte growth factors, epidermal growth factor and transforming growth factors. Each of these metabolic regulators has a distinct role at different stages in the process of hepatocyte proliferation. For example, activin A and TGF-β are noted to be key factors involved in termination of proliferation of the liver [64,65,66,67,68,69].

### 4.2. Role of microRNAs and Liver Proliferation

A number of studies have documented the importance of miRNA involvement in every step of hepatic proliferation. It has now been established that there is a biphasic pattern of miRNA regulation post-PH. Within the first 3–18 h after PH in the rat, miRNAs are up-regulated followed by a down-regulation of as many as 70% of miRNAs by 24 h. The data suggested a negative feed-back mechanism that is present between miRNAs and their genes that control this process [61,70,71,72]. In general, miRNAs are noted to accelerate or inhibit liver proliferation.

Examples of miRNAs that are associated with accelerated hepatic proliferation and regeneration include miR-21, miR-221, miR-23b, miR-122, miR-203, and miR-221 Specifically, miR-21 has been consistently observed to change during the early stages of regeneration. miR-21 knockout animal models suggest that miR-21 is responsible for cyclin D1 induction and subsequent G1 phase transition to S phase of hepatocyte cell cycle. Of interest, ursodeoxycholic acid which is a strong inducer of miR-21 appears to up-regulate miR-21 post-PH which may then lead to improved regeneration of the liver [73,74,75,76]. miR-221 is noted to inhibit hepatocytes apoptosis in mouse models, as well as delaying fulminant liver failure. It has long been observed to be up-regulated in hepatocellular carcinoma [77]. Experimental models showed that mice overexpressing miR-221 had rapid entry into the S-phase of cell cycle and up-regulation of cyclins D1, E1, A2, B1. The identified target gene for miR-221 is aryl hydrocarbon receptor nuclear translocator (Arnt) which when inhibited allowed for the promotion of hepatocyte proliferation [78,79]. miR-203 is another notable example, which epigenetically down-regulates the suppressor of cytokine signaling 3 (SOCS3), that is associated with inhibition of IL-6/STAT3 (signal transducer and activator of transcription 3). This in turn leads to promotion of hepatocyte proliferation [80,81].

Examples of miRNAs associated with inhibition of liver proliferation include: miR-26a, miR-33, miR-34a, miR-127, miR-150, and miR-378. This is achieved through a number of mechanisms that have been identified in the delay or inhibition of liver proliferation by miRNAs [76]. miR-26a appears to target cyclin D2 and E2 genes leading to impairment of proper cell cycle propagation and therefore, repression of liver regeneration. A model using adenovirus 5 (Ad5)-anti-miR26a-LUC to down-regulate miR-26a in mouse liver led to enhanced proliferation of hepatocytes and was associated with reduced liver enzymes and bilirubin after PH. The opposite was observed by using Ad5-miR26a-LUC [82]. miR-33, which is viewed as a key transcriptional factor in cholesterol biosynthesis and transportation, overexpression led to G1 cell cycle arrest and reduction of cellular proliferation [83].

miR-34a is involved in the termination phase of liver proliferation, as it is strongly induced during this period, and appears to suppress hepatocytes proliferation via inhibitory effects on potential targets such as cyclin D1, E2, CDK4, and CDK6 [84,85]. miR-127 overexpression was initially observed to suppress the growth of multiple cancers including liver [86,87]. Further studies of mouse models of liver regeneration noted that lower levels of miR-127 were observed in the initial 24 h after PH and that higher levels caused reduction in cell growth rate and cell cycle arrest in G2-M phase [88]. Two identified likely target genes were B-cell lymphoma 6 protein (Bcl6) and SET domain-containing protein 8 (Setd8). Both of these genes play a key role in cell cycle regulation and proliferative potential [89].

miR-150 down-regulation was associated with increased levels in both mRNA and vascular endothelial growth factor A (VEGF) proteins, which are expected to be elevated in the early phase of LR [90]. In humans, it was observed that patients receiving auxiliary liver transplantation had decreased tissue levels of miR-150 obtained from a liver biopsy [91]. Another key player is miR-378 which is observed to have an inverse relationship with ornithine decarboxylase (Odc1), which encodes the enzyme polyamine-synthesizing enzymes required for DNA synthesis. This ultimately led to suppression of liver proliferation [92].

It is important to note that a number of studies highlighting the role of miRNAs in liver proliferation after PH are based on animal models, and that relative abundance does not directly correlate with an anticipated role. Further studies are required to confirm post-hepatectomy changes in miRNA profile in humans and better understand their expression profile(s).

## 5. MicroRNAs and Liver Pathology

### 5.1. Hepatocellular Carcinoma

It is estimated that hepatocellular carcinoma (HCC) is the 5th most common cancer, and the 3rd cause of cancer-related deaths worldwide, with approximately 80% of HCC occurring in patients with underlying cirrhosis [93]. Certainly, the underlying liver disease as well as the chronicity of the disease, both influence the (i) risk of HCC development; (ii) rate of progression; and (iii) aggressive features of the tumor. However, there is an increasing number of studies identifying a complex network of factors and signals that influence the development and behavior of HCC. This is noted in dysregulation of cellular mechanisms that influence cell survival, fate and genome maintenance; which is a reflection of epigenetic events and cellular environmental changes [94,95].

miRNAs are gaining significant attention for their potential association with HCC, not only for the ability to act as potential biomarkers, but also as predictors of tumor behavior, and response to therapeutic targets for this multi-stage pathologic process. In addition, recent studies are evaluating their predicative role for recurrence after liver transplantation compared to available tools such as Milan criteria, and is currently estimated at 25%.

A number of studies have identified key miRNAs involved in the pathogenesis of HCC, both via down- or up-regulation of miRNA levels. For example, aggressive tumor growth and metastatic potential were found to be associated with lower levels of miR-199/b-3p and miR-122 [96,97,98]. Other studies noted that up-regulation of miR-221 and miR-222 was linked to cancer progression by enhancing cellular proliferation and migration. A number of target genes affected by dysregulated miRNAs have been identified in experimental models and observation studies both in animals and humans including the targeting of β-catenin, fibroblast growth factor receptor-1 (FGF-1), mTOR, P27, PTEN and TIMP3 tumor suppressors [99,100,101,102]. The differential expression of a number of miRNAs has been observed to be independent predictors for HCC recurrence after liver transplantation. These include miR-19a, miR-886-5p, miR-126, miR-223, miR-24 and miR 247 [103].

A recent study evaluated the use of specific miRNAs in combination of the Milan Criteria to develop a score model that may increase the accuracy for prediction of HCC recurrence. The most significant miRNAs were miR-214 and miR-3187 which allowed a cohort of patients to be classified as low and high-risk groups for recurrence [104]. This novel approach incorporated biological features of the tumor into the existing radiologic-based Milan criteria, further improving our ability to predict and manage the care of liver cancer.

### 5.2. NAFLD/NASH

Non-alcoholic fatty liver disease (NAFLD) and non-alcoholic steatohepatitis (NASH) are complex conditions that highlight the intricate relationship between the genome, and the environment. There is a worldwide increase in the prevalence of fatty liver disease, which is expected to be the leading cause of end-stage liver disease and need for liver transplantation within the next 1–2 decades. In addition, we must also recognize the parallel increase in all associated complications such as HCC and death [105]. The clinical challenges in fatty liver disease include accurate identification of patients with simple steatosis and low risk of progression to advanced liver disease *versus* those with progressive features of the disease. In addition, there is an urgent need for effective therapeutics given limitations of current strategies of life style modifications such as weight loss and exercise.

It is well established that a number of changes in miRNA expression profile is seen with NAFLD. For example, miR-34a has been noted to be significantly up-regulated both in animals and humans with features of the metabolic syndrome [106,107]. One of the main targets of miR-34a is NAD-deacetylase Sirtuin-1 (SIRT1). It is developing into a critical player involved in energy homeostasis as an activator of transcription factors Liver-X-receptor and proliferator-activated receptor-α, and an inhibitor of sterol regulatory element-binding protein 1c (SREBP1c), farnesoid X receptor (FXR) and PPAR-γ coactivator-1α [108]. In experimental models where miR-34a was silenced, successful restoration of SIRT1 and PPARα was achieved [109]. A recent study using a mouse model showed that inhibition of the p53 tumor suppressor protein resulted in reduced steatosis and lipotoxicity via a reduced effect of SIRT1 [110]. Other examples include miR-122, which was noted to be significantly down-regulated in NASH, miR-33a and miR-33b which were observed to inhibit genes involved in insulin signaling and fatty acid metabolism [111,112].

Few studies have evaluated the use of miRNA signatures as a prognostic tool. A panel of miRNAs was discovered to be approximately twice as high in healthy individuals compared to those with fatty liver disease, and they include miR-122, miR-192, miR-19a, miR-19b, miR-125, and miR-375. In this proof of concept study, miR-122 performed better than cytokeratin-18 and ALT in predicting presence and degree of fibrosis [113].

Ultimately, gaining a better understanding for the role of miRNA role in fatty liver disease would allow better-targeted therapies, whether against specific miRNAs or their target genes and mRNAs. A few studies have used antisense oligonucleotides to target mature miRNAs to reduce their biologic effect, as well as the use of viral vectors to deliver miRNA erasers, which would bind miRNA targets competitively but without producing the effect [114,115,116]. Challenges in this area include the complexity of miRNA interactions with multiple genes and targets, the optimal delivery method, and establishing the duration of therapy that is required for an effect.

### 5.3. Chronic Viral Hepatitis

Both chronic hepatitis B and C infections (HBV, HCV) have been associated with specific changes in miRNA expression profiles [117]. For example, a handful of miRNAs were noted to be down-regulated in patients with either chronic HBV or HCV infection compared to healthy controls including, miR-26a, miR-29c, miR-219 and miR-320. Furthermore, miR-122 levels showed direct correlation with HCV replication and stability. In fact, HCV RNA was reduced when miR-122 was inactivated, and increased with elevated levels of miR-122 in patients with active infection compared to healthy individuals [118,119]. Interestingly, the opposite effect was observed for miR-122 and chronic HBV infection, where observational studies noted an inverse relationship between the level of miR-122 and HBV viral DNA level. The effect appears to be mediated by inhibiting p53 mediated suppression of HBV enhancer element [120].

Of note, HCV genotype 3 has been associated with hepatic fatty accumulation, which was demonstrated by the induction of miR-27a as it targets genes involved in lipid metabolism such as transcription factor RXRα and the lipid transporter ATP-binding cassette subfamily A member 1 (ABCA1) [121].

### 5.4. Acute Liver Failure

Acute liver failure (ALF) is typically associated with massive loss of tissue, and compromise in function without evidence of underlying chronic liver disease. Given the rarity of the condition and high level of mortality without liver transplantation, there are few mechanisms known to be involved in recovery from ALF and associated hepatic proliferation. However, a recent study investigated the differential expression of miRNAs associated with liver regeneration, specifically miR-122, miR-21 and mi-221 from both serum and hepatic tissue. It was shown that those patients who had spontaneous recovery from ALF had significantly higher serum levels of all three of the aforementioned miRNAs compared to those who did not recover and either died or required liver transplantation. However, the hepatic tissue analysis showed that only miR-122 was significantly elevated in those who recovered, but not miR-221, or miR-21 [122].

### 5.5. Autoimmune Hepatitis

A recent study evaluated the expression profile of miRNAs in autoimmune hepatitis (AIH). Patients with confirmed AIH underwent microarray analysis to determine levels of miRNAs compared to healthy controls. miR-21 and miR-122 were elevated by more than 1.7 fold in the serum of untreated AIH patients; and their levels were down regulated by treatment with corticosteroids. However, there was a reduction in their levels once cirrhosis was present, and the relationship was inversely correlated with advancement of fibrosis stage [123].

### 5.6. In-born Errors of Metabolism and MicroRNAs

*Alpha-1-antitrypsin* (α1-AT) is a protein produced only by hepatocytes; and mutation of the coding gene may result in different genotypic and phenotypic manifestations, specifically MM and ZZ genotypes. α1-AT deficiency is manifest by decreased levels of circulating α1 protein leading to varying degrees of hepatic and/or pulmonary disease. A recent study identified different expression profiles of miRNAs in α1-AT deficiency patients with pulmonary disease. Specifically, miR-199-15-p was the most up-regulated miRNA in the monocytes of asymptomatic patients with the genotype ZZ compared to those with the MM genotype. Symptomatic patients with both genotypes were noted to have hypermethylation of miR-199a-2 promoter region, resulting in the inhibition of miR-199a-5p expression [124]. This highlights the potential use of miRNAs as not just markers of the disease but prognostic tools of the expected clinical course.

*Wilson’s disease* (WD) is a disorder of copper metabolism that is inherited in an autosomal recessive manner resulting from mutation of the ATP7B gene leading to cooper overload. Evidence from animal models support the role of miR-122 in predicting outcomes of severe liver injury related to copper overload. Specifically, elevated levels of miR-122 followed over time appeared to more accurately predict worsening prognosis of fulminant liver failure compared to liver enzymes, AST, ALT and bilirubin [125]. This again, highlights the importance of miRNA and their prognostic utility both in terms of sensitivity to overall, liver health, and early predictor of outcome.

*Crigler-Najjar Syndrome Type I (CN-1)* is the result of a single point mutation in the uridine 5′-diphosphate-glucuronosyltransferase 1A1 (UGT1A1) gene catalyzing liver glucuronidation of hydrophobic bilirubin into a hydrophilic isoform for excretion into bile. Mutation of the enzyme results in the accumulation of unconjugated bilirubin with catastrophic consequences including permanent mental and motor dysfunction from hyperbilirubinemia.

The animal model of CN-1 disease is the Gunn rat, which has been extensively studied for both metabolic pathways, and treatment protocols. Numerous types of gene therapy have been used to correct the hyperbilirubinemia in the rat model. Most recently, miRNAs have facilitated a better understanding and management of the condition. In one study, by including miR-142 target sequences in a lentiviral vector, the immune system response was significantly circumvented by reducing the vector expression in antigen-presenting cells. This led to normalization of hyperbilirubinemia in Gunn rats compared to those without the miR-142 sequence, highlighting the ability of miRNAs to facilitate personalized medicine [126].

## 6. Concluding Remarks

**Figure 3:**
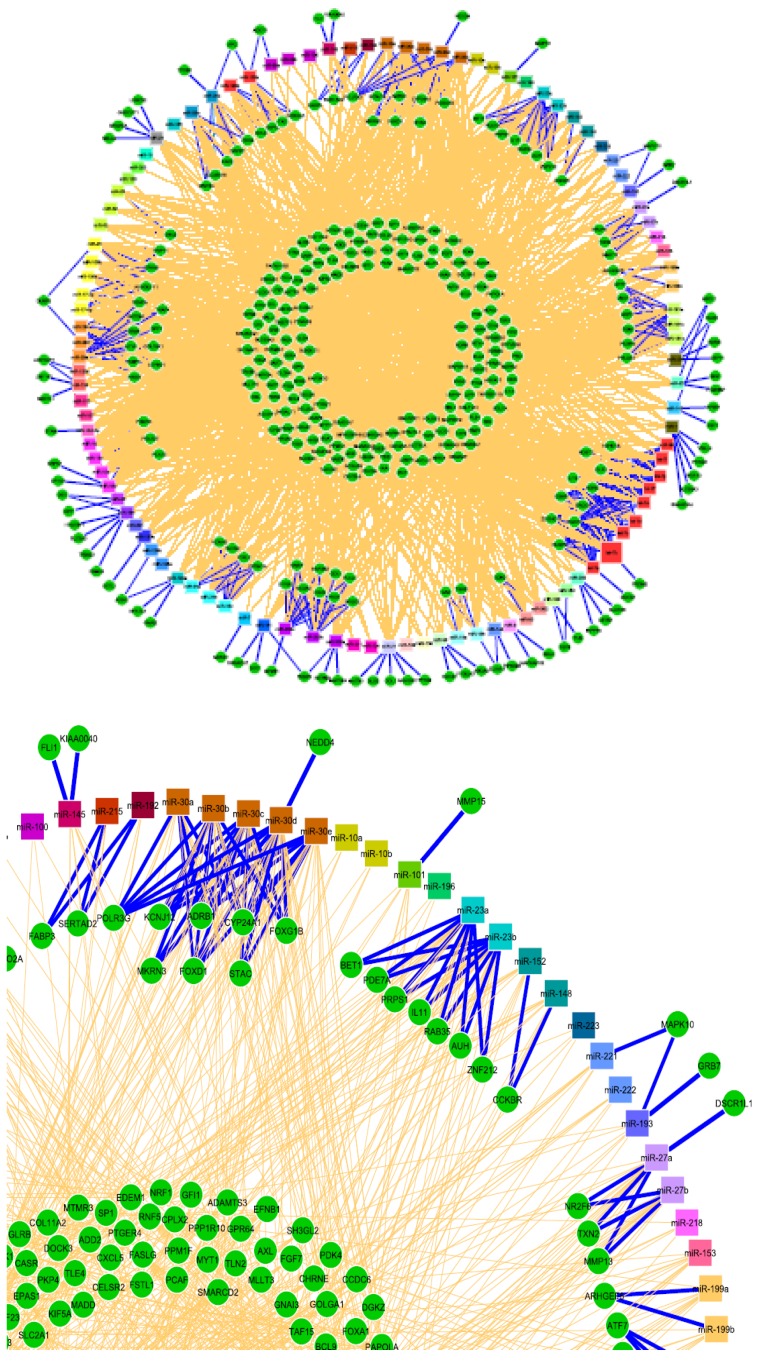
Partial illustration highlighting the complex network of interaction between microRNAs and their target genes including negative feedback mechanisms. Reprinted with permission from “Using Expression Profiling Data to Identify Human MicroRNA Targets” using GenMiR ++ data analysis algorithm (*Nat. Methods* **2007**).

The recent emergence of microRNAs as key players influencing almost every aspect of liver biology represents a milestone in our understanding and appreciation of liver health and disease. Specific changes in the expression profile of miRNAs are a reflection of the complex and delicate interaction between the genome and the environment, both globally and at the cellular level. Therefore, they offer a unique insight into the liver’s capacity and ability to face stressful events acutely and chronically. They are valuable tools as markers of disease presence, stage and response to therapy, to name just a few. Their signature profiles can be established at all stages of hepatic development from embryonic to full term; during differentiation of progenitor cells; maintenance under normal physiologic conditions; and repair/regeneration under pathologic conditions including chronic viral disease, NASH/NAFLD, and in primary liver cancers. miRNAs are dynamic regulators, differentially expressed during periods of up- and down-regulation, as well as responsive to positive and negative feedback mechanisms that provide a precise reflection of cellular activities (Figure 3).

Certainly, the application of miRNAs to personalized medicine is not without some limitations at this time. As stated, there is still more to learn and discover about miRNAs themselves as well as their interaction with each other and their target genes. It is absolutely critical to establish standardized techniques for reproducible and accurate measurements from any source. Key to our ability to translate the field of miRNAs to clinical practice is the need for more human studies in additional to the development of more accurate animal models for specific diseases.

miRNAs hold a key to the future of personalized medicine, which aims to achieve specific goals of improved assessment of disease stage and activity; accurate prediction of disease course; and better anticipation of complications. Eventually, this will lead to an individualized management plan with reduction in adverse effects, reduction in cost by avoiding unnecessary tests and therapies, and improved adherence and patient satisfaction. The future of miRNAs in the practice of medicine is both exciting and personalized.

## Figures and Tables

**Table 1:** Changes in microRNA abundance and validated target genes during liver development, regeneration and disease. Reprinted with permission from “Regulation of microRNAs and Their Role in Liver Development, Regeneration and Disease” (*Int. J. Biochem. Cell. Biol.* **2014**).

miR ^a^	Δ	Process/Disease Etiology	Model/Tissue/Cell Type	Method	Target/s	Target Validation	Reference
*let-7*	↑	Regeneration	Rat liver 3–72 h post PH	MA, qPCR	*Dicer1*	Inhibitor in Huh-7 cells with qPCR	Shu *et al.* (2011)
					*Tarbp2*		
*let-7a*	↓	Viral hepatitis (HBV)	Human HepG2 cells ± transduction HBV protein X, HCCT and NT liver	MA, qPCR	*STAT3*	Mimic/inhibitor with qPCR, W, β-Gal RA. Phenotype of target KD	Wang *et al.* (2010)
*let-7c*	↑	Development (embryo-adult)	Human embryonic (7–10 weeks) and adult liver	MA, qPCR	*TGF βR1*	Mimic/inhibitor in Huh-7 cells with mRNA/protein quantification, LA	Tzur *et al.* (2009)
1	↑	Viral hepatitis (HBV)	Transfected miR mimic into ± constitutively expressing HBV human HepG2, and Huh-7 cell line	qPCR for HBV	*HDAC4 FXRA E2F5*	Mimic in HepG2, Huh-7 cells ± constitutively expressing HBV human with qPCR, W, LA. Phenotype of target KD	Zhang *et al.* (2011)
10b	↑	NAFLD/NASH	Steatotic human L02 heps with high free fatty acid	MA, qPCR	*PPARα*	Mimic/inhibitor in L02 heps with qPCR, W, LA	Zhang *et al.* (2010)
15a	↓	Viral hepatitis (HBV)	Human HepG2 cells with overexpression or knockdown of miR-15a	Mimic/inhibitor	*HBx*	Mimic/inhibitor in HepG2 cells with LA, qPCR, W	Wang *et al.* (2013)
15b	↓	Cancer (HCC)	Human HCC tissue ± recurrence post resection	MA	*BCL-W*	Mimic/inhibitor in SNU-475 cells with W	Chung *et al.* (2010)
20a	↓	Development (embryo-adult)	Mouse foregut endoderm, hepatoblasts and adult liver	NGS	*Tgfbr2*	Inhibitor in HEK293T cells with LA and W	Wei *et al.* (2013a)
	↑	Viral hepatitis	Human HepAD38 cells ± HBV replication	qPCR, N	HBV DNA fragment	Inhibitor in HepAD38 cells with LA	Jung *et al.* (2013)
21	↑	Regeneration	Liver of rats ± Lieber-DeCarli diet for 5 weeks, assessed 1–36 h post PH	MA, qPCR	*Crebl2*	Precursor in HEK293 cells with LA	Dippold *et al.* (2012)
	↑	Regeneration (proliferation)	Mouse liver 1 h–7 days post PH	N	*Peli1*	Mimic in HEK293 cells with LA	Marquez *et al.* (2010)
	↑	Regeneration (proliferation)	Mouse liver 0–36 h post PH	qPCR	*RhoB*	Mimic/inhibitor in Hepa 1–6 cells with qPCR, W, LA	Ng *et al.* (2012)
	↑	Regeneration (proliferation)	Mouse liver 0–18 h post PH	MA, qPCR	*Btg2*	Mimic/inhibitor in Hepa 1–6 cells with qPCR, LA	Song *et al.* (2010)
	↑	Regeneration	Rat liver 3–72 h post PH	MA, qPCR	*Dicer1*	Inhibitor in Huh-7 cells with qPCR, W, LA	Shu *et al.* (2011)
	↑	Cancer (HCC)	Human T (HCC) and NT liver tissues and HCC cell lines	MA, qPCR, N	*PTEN*	Inhibitor in SK-HEP-1, SNU-182, HepG2, and PLC/PRF-5 cells with LA. Correlated target expression in tissue and cells	Meng *et al.* (2007)
22	↓	Cancer (HCC)	T (HCC) and NT human liver	qPCR	*HDAC4*	Mimic in Hep3B, SMMC-7721 cells with W, LA. Phenotype of target KD. Correlated target expression in tissue	Zhang *et al.* (2010b)
23b	↓	Regeneration (termination)	Rat liver 24–168 h post PH	qPCR	*Smad3*	Mimic in BRL-3A cells with qPCR, W, LA	Yuan *et al.* (2011)
25	↑	Cancer (CC)	Human CC and benign cell lines. T (CC) and NT human liver	qPCR	TRAIL-*DR4*	Mimic/ inhibitor in KMCH, H69, Mz-Cha-1 cells with W, LA, IF. Correlated target expression in tissue	Razumilava *et al.* (2012)
26a	↓	Regeneration (proliferation)	Mouse liver 24–168 h post PH	qPCR	*Ccnd2 Ccne2*	Mimic/inhibitor in mouse liver and Nctc-1469 cells with qPCR, W	Zhou *et al.* (2012)
	↓	Regeneration (proliferation)	Rat liver 24–72 h post PH	MA, qPCR	*Ccne2*	Mimic in HepG2 cells with qPCR, W	Chen *et al.* (2011b)
	↓	Cancer (HCC)	Mouse liver ± specific overexpression of liver-tumour initiating MYC, panel of human HCC	N, qPCR	*Ccnd2 Ccne2*	Mimic in HepG2 cells with W, LA	Kota *et al.* (2009)
	↓	Cancer (HCC)	Human T (HCC) and NT liver tissues, and ± metastasis	qPCR	*IL-6*	Mimic/inhibitor in HCC-3, MHCC97-H, HepG2 and PLC cells with LA, qPCR, Elisa	Yang *et al.* (2013)
27a	↑	Viral hepatitis (HCV)	Human HCV-compared to HBV-infected liver	qPCR	*RXRα ABCA1*	Mimic/inhibitor in Huh-7.5 cells with LA, W	Shirasaki *et al.* (2013)
27b	↑	Viral hepatitis (HCV)	Human cells (Huh-7.5) and mouse liver tissues infected with HCV	qPCR	*PPARα*	Mimic in Huh-7 cells with qPCR	Singaravelu *et al.* (2014)
29/29a	↑	Regeneration	Rat liver 3–72 h post PH	MA, qPCR	*Dicer1*	Inhibitor in Huh-7 cells with qPCR, W, LA	Shu *et al.* (2011)
	↑	Viral hepatitis (HBV)	Transgenic mouse liver, human HepG2 cells expression HBV protein X	qPCR	*PTEN*	Mimic/inhibitor in HepG2, MHCC-97L cells with qPCR, W, LA. Phenotype of target KD. Correlated target expression in HCC	Kong *et al.* (2011)
30a	↑	Development (biliary)	Mouse and human embryonic (E12.5–18.5) and adult liver. Knockdown in zebrafish	MA, N, qPCR, ISH	*Ak1 Tnrc6a*	Inhibitor in BMEL cells with MA, LA	Hand *et al.* (2009a)
34a	↑	Regeneration (termination)	Rat liver 1–9 days post PH	MA, qPCR	*Inhbb c-Met*	Mimic in BRL-3A cells with qPCR, W, LA. Phenotype of target KD.	Chen *et al.* (2011a)
	↑	ALD	Liver of mice fed 22.7–35 g/kg/day EtOH for 4 weeks, human heps, cholangiocytes and HepG2 cells ± EtOH, human ALD and paired normal liver	MA, qPCR	*CASP2 SIRT1 MMP-2 MMP-9*	Mimic in human heps with HPLC-Chip/MS analysis, qPCR, W, LA. Correlated target protein expression in heps ± EtOH.	Meng *et al.* (2012)
	↑	NAFLD/NASH	Human liver biopsy from NASH, NAFLD, weight matched normal, and lean normal subjects	qPCR	*SIRT1*	Mimic/inhibitor in Huh-7 cells with qPCR, W	Min *et al.* (2012)
	↑	NAFLD/NASH	Human liver samples from NAFLD patients with steatosis and NASH	qPCR	*SIRT1*	Precursor in primary rat heps with W, LA	Castro *et al.* (2013)
	↓	Cancer (HCC)	Human HCC tissue ± metastasis	qPCR	*c-MET*	Mimic in HepG2 cells with qPCR, W. Correlated target protein expression in liver. Phenotype of target KD	Li *et al.* (2009)
92a-1	↑	Viral hepatitis	Human HepAD38 cells ± HBV replication	qPCR, N	HBV DNA fragment	Inhibitor in HepAD38 cells with LA	Jung *et al.* (2013)
99a	↓	Cancer (HCC)	T (HCC) and NT human liver	Deep seq., qPCR	*IGF-1R mTOR*	Mimic/inhibitor in HepG2, SMMC-7721, Huh-7, HL-7702 cells, SMMC-LTNM tumor mass, with, LA, W. Correlated target expression in liver	Li *et al.* (2011)
101	↓	Cancer (HCC)	T (HCC) and NT human liver. Mouse and human liver/non-liver cells	MA, N	*MCL1*	Mimic in HEK293T cells with LA. Mimic/inhibitor in HepG2 cells with qPCR, W	Su *et al.* (2009)
122/122a	↑	Development (embryo-adult)	Mouse embryonic (E12.5–18.5) and adult liver	N, qPCR	*Cutl1*	Mimic/inhibitor in human HCC cell lines with W. Correlated target protein expression during development	Xu *et al.* (2010)
	↓	Viral hepatitis (HBV)	Liver of chronically infected HBV human patients	AP-ISH, qPCR	*CCNG1*	Mimic/inhibitor in HepG2, Huh-7 cells with LA, W. Correlated target expression to HBV load in human liver	Wang *et al.* (2012)
	↓	Cancer (HCC)	Human HCC arisen on cirrhotic livers and HCC derived cell lines	MA, N, qPCR	*CCNG1*	Precursor in SNU-449 and Hep 3B cells with LA, W. Correlated target expression in HCC	Gramantieri *et al.* (2007)
	↓	Cancer (HCC)	Mice with germline deletion of miR-122a, develop spontaneous HCC	Deletin	*KLF6*	Mimic in HEK293T cells with LA. Correlation of protein in knockout liver	Tsai *et al.* (2012)
	↓	Cancer (HCC)	Human HCC tumour and non-tumour pairs	N, qPCR	*SLC7A1 AKT3 ADAM17*	Mimic in HEK293T cells with LA	Tsai *et al.* (2009)
127	↓	Regeneration (proliferation)	Rat liver 0–168 h post PH	MA, qPCR	*Bcl6 Setd8*	Mimic/inhibitor in BRL-3A, Huh-7 cells with LA, qPCR, W. Phenotype of target KD	Pan *et al.* (2012)
129-5p	↓	Cancer (HCC)	Human T (HCC) and NT liver tissues	qPCR	*VCP*	Mimic/inhibitor in HepG2, MHCC-LM3, SK-HEP1, cells with, LA. Correlated target expression in liver	Liu *et al.* (2012)
141	↑	Viral hepatitis (HCV)	Primary human heps infected with HCV	RPA-KA	*DLC1*	Mimic/inhibitor in primary heps with qPCR, W, LA	Banaudha *et al.* (2011)
146	↑	Cancer	Human PLC/PRF/5 hepatoma cells ± IFN-α resistance	MA, qPCR	*SMAD4*	Mimic in PLC/PRF/5 cells with W. Phenotype of target KD	Tomokuni *et al.* (2011)
148a	↓	Cancer (HCC)	Human T (HCC) and NT liver ± HBV	qPCR	*HPIP*	Mimic/inhibitor in HepG2, BEL-7402, SMMC-7721, MHCC97-H and LO2 cells with WB, LA	Xu *et al.* (2013)
150	↓	Regeneration (proliferation)	Rodent liver 12–48 h post PH	qPCR	*Vegfa*	Inhibitor in primary heps with qPCR, W	Yu *et al.* (2013)
155	↑	ALD	RAW 264.7 mouse macrophages ± 50mM EtOH. Isolated Kupffer cells from mice fed Lieber-DeCarli diet (5% EtOH (*v*/*v*)) for 4 weeks	qPCR	*TNFα*	Mimic/inhibitor in RAW 264.7 cells, and inhibitor in isolated Kuppfer cells with TNFα production by ELISA	Bala *et al.* (2011)
	↑	NAFLD/NASH	Liver of mice fed choline-deficient, low methionine, amino acid-defined diet for 6–65 weeks	MA. qPCR	*Cebpβ*	Inhibitor in HepG2, Hep3B cells with qPCR, W. Correlated expression in liver	Wang *et al.* (2009)
	↑	NAFLD/NASH	Liver of mice fed lipogenic, methyl-deficient diet for 12 weeks	MA. qPCR	*Cebpβ Socs1*	Correlated protein expression with lipogenic diet by W. mimic in primary heps with W	Pogribny *et al.* (2010)
193a–3p	↑	Cancer (HCC)	Human hepatoma cell lines sensitive (QGY-7703) or resistant (SMMC-7721) to 5-fluorouracil	Deep seq. qPCR	*SRSF2*	Correlated target mRNA/protein expression with sensitive/resistant cell lines. Mimic/inhibitor in hepatoma cell lines with qPCR, W. Phenotype of target KD	Ma *et al.* (2012)
199a/b–3p		Cancer (HCC)	NT, viral infected and T (HCC) human liver	MPSS, qPCR	*PAK4*	Mimic/inhibitor in Hep3B with LA, W.	Hou *et al.* (2011)
199a–5p	↓	Cancer (HCC)	Blood from human patients with cisplatin treated un resectable/metastatic HCC. Hepatoma cell lines ± cisplatin	qPCR	*ATG7*	Mimic in HEK293T cells with LA. Mimic in Huh-7 cells with W	Xu *et al.* (2012)
200b	↑	NAFLD/NASH	Liver of mice fed lipogenic, methyl-deficient diet for 12 weeks	MA, qPCR	*Zeb1*	Correlated target protein expression with lipogenic diet. Mimic in primary mouse heps with W	Pogribny *et al.* (2010)
200c	↑	Viral hepatitis (HCV)	Chronic HCV infected human liver	MA	*FAP-1*	Mimic/inhibitor in normal human liver fibroblasts with qPCR, W	Ramachandran *et al.* (2013)
214	↓	Cancer (CC)	Human CC tissue ± metastasis	qPCR	*TWIST1*	Mimic in HEK293T cells with LA. Mimic in ICC-9810 cells with W	Li *et al.* (2012)
217	↑	ALD	Mouse AML-12 heps ± 25–100 mM EtOH for 24 h. Liver of mice fed low fat Lieber-DeCarli diet for 4 weeks	qPCR	*Sirt1*	Mimic/inhibitor in AML-12 cells with LA, qPCR, W and FTAA	Yin *et al.* (2012)
221/222	↑	Regeneration	Primary heps and mice with overexpression of miR-221	Overexpression	*Arnt*	Mimic/inhibitor in primary heps with qPCR, W, LA. Correlated target protein in post PH tissue	Yuan *et al.* (2013)
	↑	Cancer (HCC)	Human T (HCC), cirrhotic and NT liver tissues and cell lines	MA, qPCR, N	*CDKN1B (p27)*	Mimic in HeLa and HEK293T cells with W, LA. Correlated target protein in tissue samples	Pineau *et al.* (2010)
	↑	Cancer (HCC)	Human T (HCC) and NT liver tissues and cell lines	N, qPCR	*PTEN TIMP3*	Mimic/inhibitor in MEG01, H460, and Calu-1-lung cells with LA, W, qPCR.	Garofolo *et al.* (2009)
296–5p	↓	NAFLD/NASH	Human livers-obese normal, simple steatosis and NASH	qPCR	*PUMA*	Mimic/inhibitor in Huh-7, KMCH cells with qPCR, WB, LA. Correlated target mRNA and protein in human livers-normal, simple steatosis and NASH	Cazanave *et al.* (2011)
302b	↓	Development (embryo-adult)	Mouse foregut endoderm, hepatoblasts and adult liver	NGS	*Tgfbr2*	Mimic in HEK293T cells with LA and W	Wei *et al.* (2013a)
372	↑	Viral hepatitis (HBV)	HBV infected human liver, HepG2 cells ± constitutive HBV production	MA, qPCR	*NFIB*	Cluster mimic in HepG2 cells with MA. Mimic in HeLa, HepG2 cells with W, LA	Guo *et al.* (2011)
373	↑	Viral hepatitis (HBV)	HBV infected human liver, HepG2 cells ± constitutive HBV production	MA, qPCR	*NFIB*	Cluster mimic in HepG2 cells with MA. Mimic in HeLa, HepG2 cells with W, LA	Guo *et al.* (2011)
378	↓	Regeneration (proliferation)	Mouse liver 0–18 h post PH	MA, qPCR	*Odc1*	Mimic/inhibitor in Hepa 1–6 cells with qPCR, LA	Song *et al.* (2010)
467b	↓	NAFLD/NASH	Liver of mice fed high fat diet for 8 weeks. Mouse Hepa 1–6 cells ± 50 µM SFA for 24 h	qPCR	*Lpl*	Mimic/inhibitor in Hepa 1–6 cells with qPCR, W, LA. Correlated target mRNA with high fat diet and SFA treatment of heps	Ahn *et al.* (2011)
501	↑	Viral hepatitis (HBV)	Human HepG2 ± constitutive HBC production, human HBV related HCC tissue with high/low HBV replication	MA, qPCR	*HBXIP*	Inhibitor in HepG2.2.15 cells with qPCR and W	Jin *et al.* (2013)
612	↓	Cancer (HCC)	HCC tissues and paired lung metastases	MA, qPCR	*AKT2*	Mimic/inhibitor in HCCLM3 and HepG2 cells by W, LA	Tao *et al.* (2013)

^a^ Abbreviations: ALD—alcoholic liver disease; AP-ISH—alkaline phosphatase *in situ* hybridization; β-Gal RA—β-galactosidase reporter assay; CC—cholangiocarcinoma; Δ—change; Deep seq.—deep sequencing; ELISA—enzyme-linked immunosorbent assay; EtOH—ethanol; FTAA—fluorometric target activity assay; h—hours; HBV/HCV—hepatitis B/C virus; HCC—hepatocellular carcinoma; heps—hepatocytes; HPLC-Chipe/MS—high performance liquid chromatography on chip with mass spectrometry; IF—immunofluorescence; ISH—*in situ* hybridization; KD—knock down; LA—luciferase reporter assay; MA—microarray; miR—miRNA; MPSS—massively parallel sinnature sequencing; N—Northern blot; NAFLD—non-alcoholic fatty liver disease; NASH—non-alcoholic steatohepatitis; NGS—next generation sequencing; NT—non-tumourous; PH—partial hepatectomy; qPCR—quantitative reverse-transcriptase polymerase chain reaction; RPA-KA—RNA-primed array-based Klenow assay; SFA—saturated fatty acid; T—tumourous; v—volume; W—Western blot.

## Abbreviations

α1-AT	alpha-1-antitrypsin
ABCA1	ATP-binding cassette subfamily A member 1
AGO	Argonaute protein
AIH	autoimmune hepatitis
ALF	acute liver failure
Arnt	aryl hydrocarbon receptor nuclear translocator
ATP7B	ATPase copper transporting beta
Bcl 6	B-cell lymphoma 6 protein
BCR-ABL	breakpoint cluster region Abelson
BRCA 1 and 2	breast cancer genes 1 and 2
BRAF	B-Raf proto-oncogene
CN-1	Crigler-Najjar syndrome type I
DNA	deoxyribonucleic acid
FGF-1	fibroblast growth factor receptor-1
FXR	Farnesoid X receptor
HCC	hepatocellular carcinoma
HIV	human immunodeficiency virus
IL-6	interleukin-6
let-7	lethal-7 gene
LR	liver regeneration
miQPCR	open source miRNA specific qPCR platform
miRNA	micro-RNA
mTOR	mammalian target of rapamycin
NAD	nicotinamide adenine dinucleotide
NAFLD	non-alcoholic fatty liver disease
NASH	non-alcoholic steatohepatitis
NIH	National Institutes of Health
nt	nucleotide
Odc1	ornithine decarboxylase 1
P53	tumor protein 53
PH	partial hepatectomy
PPAR	peroxisome proliferator-activated receptor
PTEN	phosphatase and tensin homolog
qPCR	quantitative polymerase chain reaction
RISC	RNA-induced silencing complex
RNA	ribonucleic acid
tRNA	transfer RNA
rRNA	ribosomal RNA
siRNA	small interfering RNA
snoRNA	small nuclear RNA
Setd8	SET domain-containing protein 8
SIRT1	silent mating type information regulation 2 homolog-1
SOCS3	suppressor of cytokine signaling 3
SREBP1c	sterol regulatory element-binding protein 1c
STAT3	signal transduce and activator of transcription factor 3
TGF-beta	transforming growth factor-beta
TIMP3	tissue inhibitor of metalloproteinases 3
UGT1A1	uridine 5′-diphosphate-glucuronosyltransferase 1A1
VEGF	vascular endothelial growth factor
WD	Wilson’s disease
Wnt	wingless-related integration site
